# Role of serial cardiac ^18^F-FDG PET-MRI in Anderson–Fabry disease: a pilot study

**DOI:** 10.1186/s13244-021-01067-6

**Published:** 2021-09-06

**Authors:** Carmela Nappi, Andrea Ponsiglione, Antonio Pisani, Eleonora Riccio, Teodolinda Di Risi, Maurizio Pieroni, Michele Klain, Roberta Assante, Wanda Acampa, Emanuele Nicolai, Letizia Spinelli, Alberto Cuocolo, Massimo Imbriaco

**Affiliations:** 1 Department of Advanced Biomedical Sciences, University Federico II, Via Pansini 5, 80131 Naples, Italy; 2 Department of Public Health, University Federico II, Via Pansini 5, 80131 Naples, Italy; 3 Cardiovascular Department, San Donato Hospital, Via Pietro Nenni 22, 52100 Arezzo, Italy; 4 IRCCS-SDN, Via Emanuele Gianturco 113, 80143 Naples, Italy

**Keywords:** Anderson–Fabry disease, AFD, PET/MRI, FASTEX score

## Abstract

**Aim:**

We investigated the value of serial cardiac ^18^F-FDG PET-MRI in Anderson–Fabry disease (AFD) and the potential relationship of imaging results with FASTEX score.

**Methods and results:**

Thirteen AFD patients underwent cardiac ^18^F-FDG PET-MRI at baseline and follow-up. Coefficient of variation (COV) of FDG uptake and FASTEX score were assessed. At baseline, 9 patients were enzyme replacement therapy (ERT) naïve and 4 patients were under treatment. Two patients presented a FASTEX score of 0 indicating stable disease and did not show any imaging abnormality at baseline and follow-up PET-MRI. Eleven patients had a FASTEX score > 20% indicating disease worsening. Four of these patients without late gadolinium enhancement (LGE) and with normal COV at baseline and follow-up had a FASTEX score of 35%. Three patients without LGE and with abnormal COV at baseline and follow-up had a FASTEX score ranging from 30 to 70%. Three patients with LGE and abnormal COV at baseline and follow-up had a FASTEX score between 35 and 75%. Finally, one patient with LGE and normal COV had a FASTEX score of 100%. Of the 12 patients on ERT at follow-up, FASTEX score was significantly higher in those 4 showing irreversible cardiac injury at baseline compared to 8 with negative LGE (66 ± 24 vs. 32 ± 21, *p* = 0.03).

**Conclusion:**

^18^F-FDG PET-MRI may be effective to monitor cardiac involvement in AFD.

## Key points

- PET-MRI may be effective to monitor cardiac involvement in AFD.
- The identification of early organ involvement may influence long-term outcome.
- FASTEX score may provide assessment of systemic disease stability or progression.
- The optimal marker of reversible cardiac impairment still needs to be identified.

## Background

Anderson–Fabry disease (AFD) is a rare, X-linked, lysosomal disorder caused by mutations in the *GLA* gene encoding for the enzyme alpha-galactosidase A [1]. The consequent enzymatic deficiency causes progressive lysosomal accumulation of glycosphingolipids, in particular globotriaosylceramide (Gb3), in different cellular types and tissues [2]. Although storage occurs in several organ-specific cellular types, systemic accumulation in capillary endothelial cells has been demonstrated to play a major role in the pathological processes leading to major renal, cardiac and cerebrovascular clinical manifestations and to a significant reduction in life expectancy [3, 4]. Indeed, cardiac involvement represents the primary cause of premature mortality in AFD [5, 6]. Enzyme replacement therapy (ERT) initiation before the occurrence of irreversible organ injury determines a more favourable outcome while in patients with cardiac mature fibrosis, full-blown impaired renal function and proteinuria, the efficacy of ERT seems more limited [7]. Nevertheless, the timing of optimal ERT initiation to prevent tissue damage and organ function impairment is still debated [8, 9].

Recent studies suggested that Fabry myocardial phenotype evolves according to different disease phases [10]. There is growing evidence that inflammatory pathways are actively involved not only in myocardial AFD [11] with mature fibrosis but also at systemic level in early stages of disease [12]. In this regard cardiac simultaneous ^18^F-fluorodeoxyflucose (FDG) positron emission tomography (PET) and magnetic resonance imaging (MRI) may allow differentiation of mature fibrosis due to dense scar, from fibrosis associated with active inflammation [13, 14]. However, the role of inflammation as initial process of myocardial damage cascade and the efficacy of timely ERT on preventing irreversible disease progression remains to be fully understood. On the other hand, cardiac involvement is only one aspect of the general systemic disease progression occurring AFD. A disease stability (FASTEX) score has been recently proposed for assessment of systemic disease stability or progression [15, 16]. In the present study, we investigated the role of serial cardiac ^18^F-FDG PET-MRI in AFD and the potential relationship of imaging results with the FASTEX score.

## Methods

### Study population

We enrolled 16 consecutive patients (8 males, mean age 45 ± 13 years) with genetically proven AFD from 5 unrelated families. Exclusion criteria were pregnancy, breast-feeding and standard contraindication for MRI. Information from all patients on traditional cardiovascular risk factors and on history of AFD-associated symptoms was collected. The presence of coronary artery disease was ruled out on the basis of clinical history associated with negative stress electrocardiography or stress echocardiography. All subjects performed complete blood draw for routine biochemical. Glomerular filtration rate was estimated using the Modification of Diet in Renal Disease Study equation. For each patient, the FASTEX score was calculated [15]. This score is a mathematical model considering seven clinical parameters regarding different degrees of organ involvement in three domains: nervous, renal and cardiac system. In particular for cardiac system, echocardiography and electrocardiograph parameters are considered, in addition to the New York Heart Association class; for each domain, a scoring system ranging from 0 (no damage) to 4 (severe damage) is used. The weighted variation of these parameters in the three domains, across two different temporal points, is converted in percentage accounting for nervous, renal and cardiac severity score change. The sum of each severity domain corrected by their interaction results is the individual FASTEX score [15]. The present study included most of patients described in a previous paper [13] in which only baseline ^18^F-FDG PET-MRI findings were described. The study conformed to the principles outlined in the Declaration of Helsinki and was approved by the local Ethics Committee of our Institution, and all patients were informed and signed a written consent to participate to this study.

### Imaging

In all patients, cardiac ^18^F-FDG PET-MRI (Biograph mMR; Siemens Healthcare, Erlangen, Germany) was performed according to the Society of Nuclear Medicine and Molecular Imaging and the American Society of Nuclear Cardiology guidelines [16]. Subjects were instructed to consume 2 high-fat, low-carbohydrate meals the day before the study and then fasted for at least 6 h before the scan in order to ensure adequate suppression of ^18^F-FDG uptake [17]. Before imaging, patients were asked regarding adequate adherence to diet. Each patient was intravenously injected with 370 MBq of ^18^F-FDG and imaging was performed 45 min later [18]. A single-bed-position PET emission scan was acquired over 20 min simultaneously with a whole heart cardiac MRI balanced steady state free precession cine sequence and T2-weighted short tau inversion recovery (STIR). Cardiac MRI was triggered by ECG. Subsequently, late gadolinium enhancement (LGE) inversion recovery sequences were obtained 10 min after administration of a 0.1-mmol/kg-body weight Gadobutrol (Gadovist, Bayer Schering Pharma, Berlin, Germany) bolus.

### PET-MRI analysis

A radiologist and a nuclear medicine physician in consensus evaluated cardiac ^18^F-FDG PET-MRI images on a dedicated workstation, as previously described [13]. Left ventricular (LV) volumes, mass, ejection fraction, wall thickness and LGE patterns were analysed. According to ^18^F-FDG uptake pattern, PET images were classified in homogeneous, heterogeneous and focal. Only focally increased cardiac uptake was considered a positive finding for the presence of active cardiac inflammation [13, 17]. The standardised uptake value (SUV) was also calculated. The intensity of ^18^F-FDG uptake was assessed by measuring the SUV in 17 myocardial segments and the average SUV and standard deviation (SD) of the SUV were quantified for each patient. SUV was automatically calculated by Corridor 4DM v.7.0 software from Invia Medical Imaging Solutions (Ann Arbor, Michigan) with a threshold of 50%. The coefficient of variation (COV) of the SUV in each patient was calculated as the SUV SD divided by the average SUV as an index of heterogeneity of ^18^F-FDG uptake [14, 17]. SUV has been blindly measured by two nuclear medicine physicians, and data were averaged. The intra-observer and inter-observer variability of SUV measurements were < 5%. As previously reported, control subjects, with no evidence of active inflammatory, coronary or valvular diseases, or diabetes mellitus or severe hepatic, renal, malignant, and hematologic diseases and not receiving corticosteroids, demonstrated a mean COV and SD values of 0.12 and 0.025, respectively, suggesting to distinguishing physiological from abnormal FDG uptake according to a COV cut-off value > 0.17 [14, 19]. A focal increase of FDG uptake with COV > 0.17 was considered a positive finding for the presence of active cardiac inflammation [13, 17]. Both LGE and T2-weighted STIR images were compared to PET images using source data and fused images according to 17-segment model of LV.

### Statistical analysis

Continuous variables are expressed as mean ± SD and categorical data as percentages. For comparison of groups, unpaired t test was performed. A *p* value < 0.05 was considered statistically significant. Statistical analyses of all data was performed using SPSS software (SPSS 21.0 for Windows, IBM, Chicago, IL, USA).

## Results

From an initial population of 16 patients with AFD, 3 patients (2 with evidence of LGE, positive STIR findings and abnormal COV and 1 patient with only LGE at baseline) implanted cardioverter-defibrillator for the occurrence of arrhythmic events following baseline examination and did not perform follow-up ^18^F-FDG PET-MRI. Hence, 13 patients (8/13, 62% males) underwent baseline and follow-up cardiac ^18^F-FDG PET-MRI (mean follow up time: 58 ± 13 months). The clinical characteristics of the overall population at baseline and follow-up are shown in Table 1. No patient had diabetes mellitus or coronary artery disease. While only 4/13 patients were receiving ERT at baseline scan (31%), most patients (12/13) were under ERT at follow-up (92%).

**Table 1:** Clinical characteristics of the overall population at baseline and follow-up

	Baseline study	Follow-up study
Patients (*n*)	16	13
Age (years)	45 ± 13	50 ± 14
Male gender, *n* (%)	8 (50)	6 (62)
Weight (kg)	68 ± 12	70 ± 11
Hypertension, *n* (%)	5 (31)	5 (38)
Smoking, *n* (%)	1 (6)	1 (8)
Classic Anderson–Fabry disease, *n* (%)	13 (81)	10 (77)
Late onset Anderson–Fabry disease, *n* (%)	3 (19)	3 (23)
Enzyme replacement therapy, *n* (%)	6 (37)	12 (92)
Left ventricular mass (g)	58 ± 23	54 ± 8
Left ventricular end-diastolic volume (mL)	77 ± 19	76 ± 7
Left ventricular end-systolic volume (mL)	26 ± 10	29 ± 2
Left ventricular ejection fraction (%)	67 ± 7	63 ± 7

Values are expressed as mean value ± standard deviation or as number (percentage) of subjects

### Baseline and follow-up imaging findings

Baseline and follow-up imaging results according to ERT are reported in Table 2. At baseline, 9 patients (#1–9) were ERT naïve and 4 patients (#10–13) were already under therapy.

**Table 2:** Imaging findings in the 13 patients undergoing baseline and follow-up ^18^F-FDG PET-MRI

Patient	Sex	Age	ERT		LGE		STIR		LVH		COV		FASTEX score
			Baseline	Follow-up	Baseline	Follow-up	Baseline	Follow-up	Baseline	Follow-up	Baseline	Follow-up	
1	F	33	−	+	−	−	−	−	−	−	−	−	35
2	M	19	−	+	−	−	−	−	−	−	−	−	35
3	M	45	−	+	−	−	−	−	+	+	−	−	35
4	M	53	−	+	−	−	−	−	+	+	−	−	35
5	F	25	−	+	−	−	−	−	−	−	−	−	0
6	F	66	−	−	−	−	−	−	−	−	−	−	0
7	F	50	−	+	−	−	−	−	−	−	+	−	50
8	M	36	−	+	−	−	−	−	−	−	+	−	70
9	M	41	−	+	+	+	+	+	+	+	+	+	35
10	M	32	+	+	−	−	−	−	−	−	+	-	30
11	F	45	+	+	+	+	−	−	−	−	+	+	55
12	F	50	+	+	+	+	+	+	+	+	+	+	75
13	F	63	+	+	+	+	−	-	+	+	−	−	100

*ERT* enzyme replacement therapy*, LGE* late gadolinium enhancement, *STIR* short tau inversion recovery, *LVH* left ventricular hypertrophy, *COV* coefficient of variance

Among the 8 patients without LGE at baseline that were not receiving ERT, 6 had normal COV and 2 abnormal COV with focal pattern of increased uptake in the infero-lateral region of the LV. At follow-up scan, negative PET-MRI findings were observed in all 8 patients, including those demonstrating focal pattern of increased ^18^F-FDG-uptake at baseline, who started ERT according to clinical judgement. Of note, all 6 patients who did not show LV hypertrophy at baseline, still showed normal LV wall thickness. Only one ERT naïve patient (#9) showed cardiac damage at baseline, including LGE, LV hypertrophy as well as abnormal COV confirmed at follow-up study. Finally, one patient (#6) remained ERT naïve, showing normal PET-MRI results at both baseline and follow-up studies.

Of the 4 patients already under ERT at baseline, one (patient #10) showed negative MRI and focal pattern of increased ^18^F-FDG uptake at baseline and negative ^18^F-FDG PET-MRI findings at follow-up, 2 (patients #11 and 12) demonstrated abnormal COV in presence of LGE at both imaging scans, and one (patient #13) had mature fibrosis at baseline with no sign of inflammation at PET imaging, confirmed at follow-up. Concordance between COV and LGE was observed in a 9/13 (69%) patients at baseline and in 12/13 (92%) patients at follow-up.

**Fig. 1:**
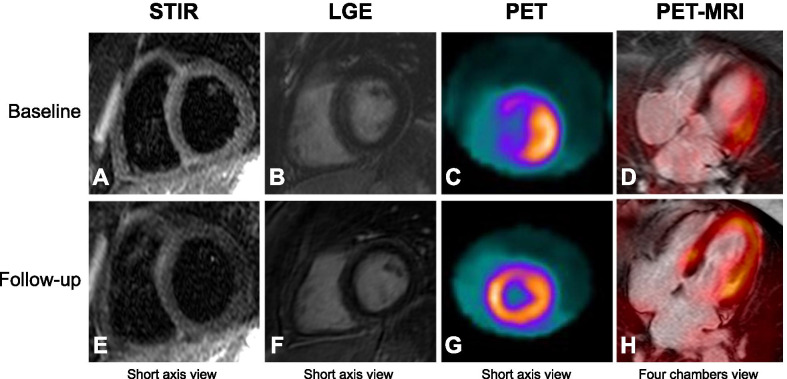
50-year-old female (patient #7) with negative STIR (**A**), no LGE (**B**) and increased ^18^F-FDG uptake in the lateral region of the LV (**C**) and corresponding fused images (**D**) at baseline study. Unremarkable PET-MRI findings at follow-up study (**E**–**H**)

**Fig. 2:**
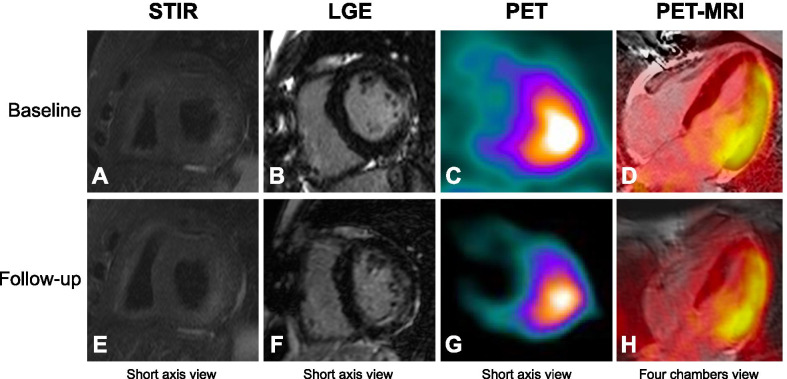
50-year-old female (patient #12) with focal hyperintensity on STIR image (**A**) and focal LGE in the basal segments of the lateral wall of the LV (**B**), increased ^18^F-FDG uptake in the corresponding myocardial region (**C**) and relative fused images (**D**) at baseline study. No significant changes of PET-MRI findings at follow-up study (**E**–**H**)

**Fig. 3:**
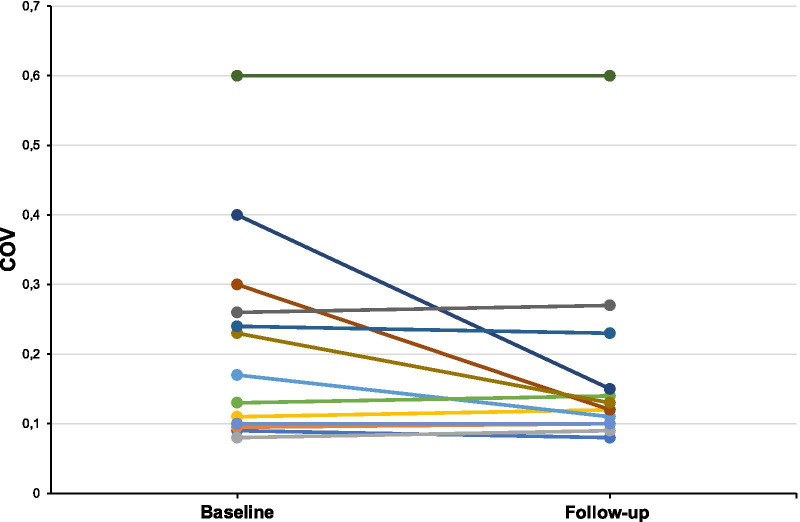
Individual COV values at baseline and follow-up

Representative images are shown in Figs. 1 and 2. For a better understanding of the evolution of cardiac involvement from baseline to follow-up, individual PET imaging findings at each scan are depicted in Fig. 3.

### Relationship between imaging findings and FASTEX score

The FASTEX score of each patient is reported in Table 2. Only 2 patients presented a FASTEX score of 0, indicating overall stable disease. Of note, these 2 patients did not show any imaging abnormality at both baseline and follow-up studies. The remaining 11 patients had a FASTEX score > 20% indicating global disease worsening. In particular, patients 1, 2, 3 and 4 without LGE, with negative STIR and normal COV at baseline and follow-up scans had a FASTEX score of 35%. Three patients with normal MRI findings and abnormal COV at baseline and follow-up studies had a FASTEX score of 50% (patient #7), 70% (patient #8) and 30% (patient #10). Two patients with abnormal MRI and PET findings at baseline and follow-up had a FASTEX score of 35% (patient #9) and 75% (patient #12). Patient 11 with LGE, negative STIR and abnormal COV at both imaging studies had a FASTEX score of 55%, whereas patient 13 with LGE and negative STIR and normal COV presented a FASTEX score of 100%. Moreover, of the 12 patients on ERT at the time of follow-up evaluation the FASTEX score was significantly higher in the 4 patients already showing irreversible cardiac injury at baseline scan compared to the 8 patients with negative LGE (66 ± 24 vs. 32 ± 21, *p* = 0.03).

## Discussion

This is the first study investigating the role of serial hybrid cardiac ^18^F-FDG PET-MRI in patients with AFD. We observed that among the 8 ERT naïve subjects with no sign of mature fibrosis at baseline ^18^F-FDG PET-MRI, those 2 with evidence of focal ^18^F-FDG uptake after starting ERT showed normal ^18^F-FDG PET-MRI findings at follow-up imaging. These data suggest that disease course had been stabilized by treatment before the onset of irreversible cardiac damage. Similarly, in the remaining 6 patients with normal ^18^F-FDG PET-MRI findings at baseline, no evidence of cardiac involvement was observed at follow-up, during ERT.

In the 2 patients already under ERT at baseline examination with cardiac damage, identified by evidence of focal LGE as expression of myocardial fibrosis and increased ^18^F-FDG uptake as expression of active inflammatory process, ^18^F-FDG PET-MRI findings were confirmed at follow-up, underlining the limited benefit obtained from treatment, when injury has been already established, including persistence of inflammatory pattern of FDG uptake.

Moreover, of the 12 patients on ERT at the time of follow-up evaluation the FASTEX score, that provides information on the disease progression, was significantly higher in those 4 already showing irreversible cardiac injury at baseline scan compared to 8 with negative LGE, underlining that stabilisation of disease may be more successful in patients starting ERT before the onset of organ damage. It should be noted that while normal COV values were always associated with negative STIR at baseline and follow-up imaging, negative STIR results did not predict neither COV changes nor normal COV findings, demonstrating disagreement in four patients at baseline and in one patient at follow-up. This finding should take into consideration that, although FDG uptake suggests activation of inflammatory cells with potential myocardial oedema, STIR sequence may not be the optimal tool to look for subtle oedema in AFD.

The importance of early and correct diagnosis is essential in patients with AFD as well as the need to identify non-invasive biomarkers able to detect early cardiac involvement, before the onset of irreversible myocardial fibrosis, and potentially influence treatment response. It is also widely recognised that early ERT administration, especially in the pre-hypertrophic phase, prevents progression of the disease, thus influencing patient’s outcome. In particular, Imbriaco et al. [14] verified the possibility of identifying different stages of AFD related cardiac disease involvement and progression from early phases to more advanced disease by hybrid ^18^F-FDG PET-MRI. The authors described a potential association between myocardial inflammation, as documented by abnormal ^18^F-FGD uptake findings, and glycosphingolipid burden accumulation with interstitial fibrosis advancement, as recognised by pseudo-normalisation of abnormal T1 values. On the light of irreversible myocardial damage and fibrosis development prevention, the identification of this intermediate stage of disease may allow an early and more effective therapeutic approach.

The role of focal ^18^F-FDG uptake in AFD-related cardiac involvement progression has been also previously investigated in females with α-galactosidase A mutation. Spinelli and coworkers [19] showed the association between impaired LV longitudinal function and abnormal ^18^F-FDG findings as an early sign of myocardial injury, confirming the potential role of inflammation in glycosphingolipids storage disorders. The emerging concept of myocardial inflammation coexisting with myocardial fibrosis in patients with AFD has been proposed in several prior studies [11, 20–22]. More recently, Nordin et al. [10] suggested a model of myocardial phenotype evolution in AFD. From the initial glycosphingolipids’ accumulation, myocardial damage occurs through hypertrophy and inflammatory phase ending up in fibrosis with irreversible cardiac impairment. The initial storage phase starts in childhood and is sub-clinical; in this phase, T1 mapping values are low and are associated with normal LV mass values. In the myocyte hypertrophy and inflammation phase, LGE and inflammation appears mainly in the infero-lateral wall and are associated with elevation of chronic troponin, without evidence of LV wall thinning. In the late and irreversible fibrotic phase, persistent LVH and troponin elevation is present, in association with myocardial cell death and LV wall thickening. Camporeale et al. [23] also demonstrated that in pre-hypertrophic Fabry disease, the presence of low T1 values is a risk factor for disease worsening, thus representing a potential new tool in prognostic stratification and therapeutic approach. More recently, Augusto et al. [21] using a combination of blood and cardiac MRI biomarkers have shown that when LGE is present in AFD patients, it is strongly associated with high T2-weighted values, suggestive of myocardial oedema, and chronic troponin elevation. This oedema has prognostic significance and determines baseline cardiac electromechanical changes and clinical worsening after 1 year, suggesting a potential new treatment strategy. Frustaci et al. [24] first documented histological evidence of myocarditis in 56% of a large sample of patients with AFD undergoing endo-myocardial biopsy. These findings are consistent with hypothesis that if on the one hand myocarditis may develop due to interstitial damage through inflammatory cell infiltration, oedema, and cell necrosis, on the other it may also trigger myocardial fibrosis evolution through the activation of transforming growth factor b1. The same group recently examined the explanted heart from a 57-year-old man with AFD cardiomyopathy on three years ERT, presenting with ventricular fibrillation [25]. The authors reported a severe virus-negative myocarditis, with extensive inflammation involving cardiomyocytes, coronary vessels, conduction tissue and cardiac ganglions and hypothesising a Gb3-induced auto-reactive myocarditis, as a possible cause of ERT resistance and irreversible cardiac impairment. The role of ^18^F-FDG-PET in myocardial inflammation identification has also been largely investigated. In particular, Nensa et al. [26] prospectively compared ^18^F-FDG-PET to LGE and T2-weighted MRI sequences, using hybrid ^18^F-FDG-MRI in patients with suspected myocarditis, demonstrating an overall good agreement between MRI findings and that abnormal myocardial ^18^F-FDG PET uptake. Given the high levels of glucose transporters (in particular GLUT1 and GLUT3) and hexokinase activity expressed by all cells of the monocyte/macrophage family and lymphocytes, glucose uptake evaluation is hallmark of inflammation enabling^18^F-FDG PET imaging to directly quantify the metabolic activity of inflammatory cell infiltrates.

The main limitation of our study is represented by the small sample size, but it should be considered that AFD is a rare disorder and available data on prognostic stratification are very limited. Moreover, our findings refer to a single-centre experience. Hence, as future perspective, our results may encourage a multi-centre investigation paving the way for a deeper AFD insight. However, due to radiation exposure the correct timing for follow-up imaging still needs to be defined. A clear algorithm for establish role and timing of performing ^18^F-FDG PET-MRI should take into account the average exposition that is around 8 mSv for a median dose of 370 MBq of ^18^F-FDG, while the natural background ranges from about 1.5 to 3.5 mSv. Further, it should be considered that naïve patients may mostly benefit from ^18^F-FDG PET-MRI evaluation. With regard to imaging protocol, the use of T1 and T2 mapping and extra-cellular volume estimation would have been an added value to guide patients’ treatment and monitoring strategies and should be taken into account for further investigations. Further, endo-myocardial biopsy data are not available. However, the value of biopsy as gold standard is limited due to low sensitivity attributable to sampling errors to the point that LGE identification and localisation usually guides biopsy [27].

## Conclusions

FASTEX score, indicating systemic disease worsening, is lower in patients who started ERT in absence of cardiac fibrosis at baseline scan, highlighting greater benefits of early ERT initiation, before irreversible damage occurs. However, the optimal marker of reversible cardiac impairment onset still needs to be identified. ^18^F-FDG PET-MRI can be further explored as a multimodality imaging tool to follow-up cardiac involvement in AFD at early stage, to start ERT in a timely fashion, and to monitor disease progression, thus improving patients’ outcome.

## Data Availability

The datasets used and/or analysed during the current study are available from the corresponding author on reasonable request.

## Abbreviations

AFD: Anderson–Fabry disease
COV: Coefficient of variation
ERT: Enzyme replacement therapy
FDG: Fluorodeoxyglucose
FU: Follow-up
Gb3: Globotriaosylceramide
LGE: Late gadolinium enhancement
LV: Left ventricular
MRI: Magnetic resonance imaging
PET: Positron emission tomography
SD: Standard deviation
STIR: T2-weighted short tau inversion recovery
SUV: Standardised uptake value

## References

[1] Zarate YA, Hopkin RJ (2008). Fabry’s disease. Lancet.

[2] Brady RO, Gal AE, Bradley RM (1967). Enzymatic defect in Fabry’s disease. N Engl J Med.

[3] Schiffmann R, Warnock DG, Banikazemi M (2009). Fabry disease: progression of nephropathy, and prevalence of cardiac and cerebrovascular events before enzyme replacement therapy. Nephrol Dial Transplant.

[4] Wilcox WR, Oliveira JP, Hopkin RJ (2008). Females with Fabry disease frequently have major organ involvement: lessons from the Fabry Registry. Mol Genet Metab.

[5] Weidemann F, Linhart A, Monserrat L, Strotmann J (2010). Cardiac challenges in patients with Fabry disease. Int J Cardiol.

[6] Linhart A, Kampmann C, Zamorano JL (2007). Cardiac manifestations of Anderson–Fabry disease: results from the international Fabry outcome survey. Eur Heart J.

[7] Arends M, Biegstraaten M, Hughes DA (2017). Retrospective study of long-term outcomes of enzyme replacement therapy in Fabry disease: analysis of prognostic factors. PLoS One.

[8] Kampmann C, Perrin A, Beck M (2015). Effectiveness of agalsidase alfa enzyme replacement in Fabry disease: cardiac outcomes after 10 years’ treatment. Orphanet J Rare Dis.

[9] Weidemann F, Niemann M, Störk S (2013). Long-term outcome of enzyme-replacement therapy in advanced Fabry disease: evidence for disease progression towards serious complications. J Intern Med.

[10] Nordin S, Kozor R, Medina-Menacho K (2019). Proposed stages of myocardial phenotype development in Fabry disease. JACC Cardiovasc Imaging.

[11] Nordin S, Kozor R, Bulluck H (2016). Cardiac Fabry disease with late gadolinium enhancement is a chronic inflammatory cardiomyopathy. J Am Coll Cardiol.

[12] Augusto JB, Moon JC (2019). Mapping phenotype development in Fabry disease. Circ Cardiovasc Imaging.

[13] Nappi C, Altiero M, Imbriaco M (2015). First experience of simultaneous PET/MRI for the early detection of cardiac involvement in patients with Anderson–Fabry disease. Eur J Nucl Med Mol Imaging.

[14] Imbriaco M, Nappi C, Ponsiglione A (2019). Hybrid positron emission tomography-magnetic resonance imaging for assessing different stages of cardiac impairment in patients with Anderson–Fabry disease: AFFINITY study group. Eur Heart J Cardiovasc Imaging.

[15] Mignani R, Pieruzzi F, Berri F (2016). FAbry STabilization indEX (FASTEX): an innovative tool for the assessment of clinical stabilization in Fabry disease. Clin Kidney J.

[16] Dorbala S, Di Carli MF, Delbeke D (2013). SNMMI/ASNC/SCCT guideline for cardiac SPECT/CT and PET/CT 1.0. J Nucl Med.

[17] Osborne MT, Hulten EA, Murthy VL (2017). Patient preparation for cardiac fluorine-18 fluorodeoxyglucose positron emission tomography imaging of inflammation. J Nucl Cardiol.

[18] Dilsizian V, Bacharach SL, Beanlands RS (2016). ASNC imaging guidelines/SNMMI procedure standard for positron emission tomography (PET) nuclear cardiology procedures. J Nucl Cardiol.

[19] Spinelli L, Imbriaco M, Nappi C (2018). Early cardiac involvement affects left ventricular longitudinal function in females carrying α-galactosidase A mutation. Circ Cardiovasc Imaging.

[20] Nordin S, Kozor R, Vijapurapu R (2019). Myocardial storage, inflammation, and cardiac phenotype in Fabry disease after one year of enzyme replacement therapy. Circ Cardiovasc Imaging.

[21] Augusto JB, Nordin S, Vijapurapu R (2020). Myocardial edema, myocyte injury, and disease severity in Fabry disease. Circ Cardiovasc Imaging.

[22] Hayashi Y, Hanawa H, Jiao S (2015). Elevated endomyocardial biopsy macrophage-related markers in intractable myocardial diseases. Inflammation.

[23] Camporeale A, Pieroni M, Pieruzzi F (2019). Predictors of clinical evaluation in prehypertrophic Fabry disease. Circ Cardiovasc Imaging.

[24] Frustaci A, Verardo R, Grande C (2018). Immune-mediated myocarditis in Fabry disease cardiomyopathy. J Am Heart Assoc.

[25] Frustaci A, Scarpa M, Da Riol RM (2020). Fabry cardiomiopathy: Gb3-induced auto-reactive panmyocarditis requiring heart transplantation. ESC Heart Fail.

[26] Nensa F, Kloth J, Tezgah E (2018). Feasibility of FDG-PET in myocarditis: Comparison to CMR using integrated PET/MRI. J Nucl Cardiol.

[27] Mahrholdt H, Goedecke C, Wagner A (2004). Cardiovascular magnetic resonance assessment of human myocarditis: a comparison to histology and molecular pathology. Circulation.

